# Fractal Analysis and the Diagnostic Usefulness of Silver Staining Nucleolar Organizer Regions in Prostate Adenocarcinoma

**DOI:** 10.1155/2015/250265

**Published:** 2015-08-20

**Authors:** Alex Stepan, Cristiana Simionescu, Daniel Pirici, Raluca Ciurea, Claudiu Margaritescu

**Affiliations:** ^1^Department of Pathology, University of Medicine and Pharmacy of Craiova, Petru Rares Street 2, Dolj, 200349 Craiova, Romania; ^2^Department of Research Methodology, University of Medicine and Pharmacy of Craiova, Petru Rares Street 2, Dolj, 200349 Craiova, Romania

## Abstract

Pathological diagnosis of prostate adenocarcinoma often requires complementary methods. On prostate biopsy tissue from 39 patients including benign nodular hyperplasia (BNH), atypical adenomatous hyperplasia (AAH), and adenocarcinomas, we have performed combined histochemical-immunohistochemical stainings for argyrophilic nucleolar organizer regions (AgNORs) and glandular basal cells. After ascertaining the pathology, we have analyzed the number, roundness, area, and fractal dimension of individual AgNORs or of their skeleton-filtered maps. We have optimized here for the first time a combination of AgNOR morphological denominators that would reflect best the differences between these pathologies. The analysis of AgNORs' roundness, averaged from large composite images, revealed clear-cut lower values in adenocarcinomas compared to benign and atypical lesions but with no differences between different Gleason scores. Fractal dimension (FD) of AgNOR silhouettes not only revealed significant lower values for global cancer images compared to AAH and BNH images, but was also able to differentiate between Gleason pattern 2 and Gleason patterns 3–5 adenocarcinomas. Plotting the frequency distribution of the FDs for different pathologies showed clear differences between all Gleason patterns and BNH. Together with existing morphological classifiers, AgNOR analysis might contribute to a faster and more reliable machine-assisted screening of prostatic adenocarcinoma, as an essential aid for pathologists.

## 1. Introduction

Prostate cancer is considered the second cause of death by malignant neoplasia in the male population around the world, over 95% of all diagnosed cases being represented by acinar adenocarcinoma [1–3]. The incidence of prostate cancer in Romania in 2012 was officially estimated at 20 cases per 100,000 males, these low incidence rates being largely due to underregistration of prostate cancer, as well as the lack of sensitive diagnostic tests for an early detection [4, 5].

Pathological diagnosis of prostate neoplasia is sometimes cumbersome and the differential diagnosis needs to be made with atypical benign lesions. In these cases, techniques such as immunohistochemistry for acinar basal cells [6, 7], the histochemical silver staining for the nucleolar organiser regions (AgNORs) [8], and genetic testings have brought an invaluable support in establishing the correct diagnosis [9, 10]. AgNOR silver impregnation protocols have been utilized and standardized [8, 11, 12] for counting and morphometry and may contribute to the differential diagnosis between benign and malignant prostate lesions, either alone [13–16] or in combination with immunohistochemistry and serologic markers [17–19], and have even been assessed as a prognostic factor for this pathology [20, 21]. Nucleolar organizing regions (NORs) represent fragments of ribosomal DNA involved in transcription of ribosomal RNA, which due to their association with nonhistonic argyrophilic proteins may be observed and quantified after precipitation of silver nitrate [8, 22].

AgNOR analysis is justified by the well-known morphological changes of nucleoli in prostate adenocarcinoma [6]. Beyond subjective observations, automated image analysis for diagnostic applications is currently a dynamically evolving domain, supporting an increasing standardization and an accuracy of the diagnostic process [23, 24]. While classical objective morphological denominators like areas and diameters have proved insufficient to describe highly variable and complex pathological processes, scale-invariant parameters like fractal dimension (FD) have been very useful in characterizing complex and nonregular objects [25]. Classical morphological features are based on Euclidean geometric system that has three dimensions as integers, while the FD of an object is a real (adimensional) number that expresses the morphological complexity and the inner self-similarity of the object or, in simple terms, it characterizes the space-filling properties of that object [25]. The closer this dimension is to the topological dimension of the space in which it resides, the greater its space-filling capacity is, and thus its FD value, with a bidimensional structure (like planar images) having FD values between 1 and 2.

This complexity-related concept is now widely used in pathology to describe tumor angiogenesis, chromatin distribution in malignant cells, or even prostate glands' morphology [26–28].

In this context, the advent of more powerful image analysis segmentation algorithms based on color, intensity, texture, and background contrast, coupled with fractal analysis of AgNOR regions might offer supplemental valuable classifiers for future machine-based diagnostic algorithms that will help the pathologist with classifying benign, atypical, and malignant prostate lesions.

## 2. Methods

### 2.1. Patients

Formalin-fixed paraffin-embedded archived prostate transurethral resection of the prostate (TURP) biopsies were selected from previously confirmed patients with benign nodular hyperplasia (number (*N*) = 8), atypical adenomatous hyperplasia (AAH) (*N* = 5), and Gleason grading of 2 (*N* = 5), 3 (*N* = 5), 4 (*N* = 7), and 5 (*N* = 9) conventional acinar adenocarcinoma. All selected cases belong to the archive of the Pathology Department from the Emergency County Hospital 1, Craiova, Romania, and were diagnosed without equivoque as belonging to the respective groups (Alex Stepan, Claudiu Margaritescu, and Daniel Pirici), following the latest WHO grading system [2]. All patients that have been included were at their first presentation, thus without any treatments. A written informed consent was obtained for each patient from their relatives, accepting tissue sampling for research purposes, and the study was approved by the responsible ethical committee.

### 2.2. Immunohistochemistry and AgNOR Staining

In order to stain for the nucleolar organizers and still identify the histopathology of the tissue with the best contrast, we have optimized a mixed protocol combining the silver staining protocol as proposed by the International Committee on AgNOR Quantitation with immunohistochemistry for basal cells and a Nuclear Red counter staining [12].

The slides were first dewaxed in xylene and rehydrated through graded alcohols to distilled water, and then antigen retrieval was performed by microwaving the sections in sodium-citrate buffer (0.01 M sodium-citrate monohydrate, pH 6.0) for 20 minutes at 650 W. After cooling, the sections were incubated for 30 minutes in a 1% hydrogen peroxide solution and then blocked for 1 hour in 3% skim milk (Bio-Rad, Medicalkit, Craiova, Romania). A mix of 34*β*E12 (1 : 100) and p63 (1 : 200) mouse anti-human primary antibodies (Dako, Redox, Bucharest, Romania) was added onto the slides for 18 hours at 4°C; the next day the signal was amplified utilizing a peroxidase polymer-based system (Nichirei-Histofine, Medicalkit, Craiova, Romania), and then the signal was detected with Permanent HRP Green substrate (Zytomed, Medicalkit, Craiova, Romania). After washing the slides in distillated water, a modified silver staining protocol was performed [12]. The slides were counterstained in 0.1% Nuclear Red prepared in a 5% aqueous aluminum sulphate solution. The slides were dehydrated fast, cleared in xylene, and mounted in DPX (Fluka, Medicalkit, Craiova, Romania).

### 2.3. Image Grabbing and Analysis

**Figure 1:**
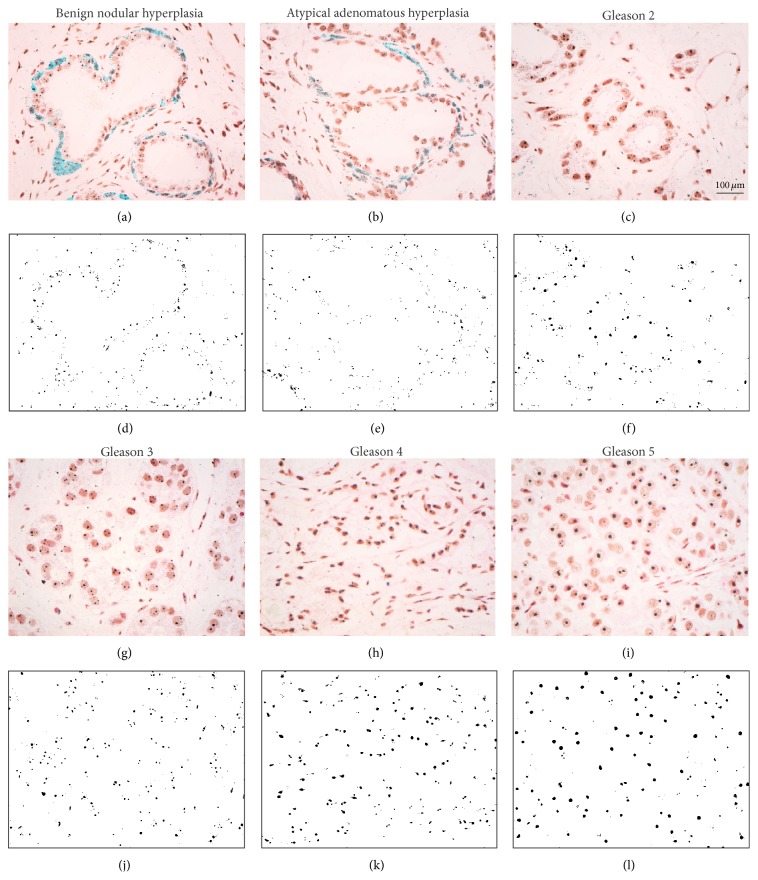
Segmentation and binarisation of argyrophilic nucleolar organizer regions (AgNORs). Histochemical-immunohistochemical stainings have been performed for AgNORs (dark dots) and glandular basal cells (a cocktail of p63 and 34*β*E12, visualized in green), with a Nuclear Red counterstaining in order to ensure a good pathological classification of nodular benign nodular hyperplasia (a), atypical adenomatous hyperplasia (b), and acinar adenocarcinoma Gleason grades 2 (c), 3 (g), 4 (h), and 5 (i). Following segmentation, binary images of AgNORs substractions have been generated ((d)–(f) and (j)–(l)). Scale bar represents 100 *μ*m.

**Figure 2:**
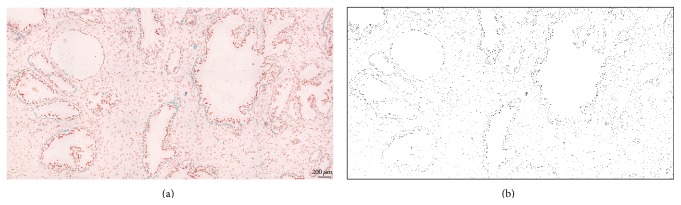
Larger scan areas (24x 400x) utilized for segmentation and binarisation of argyrophilic nucleolar organizer regions (AgNORs). An area of benign nodular hyperplasia is shown ((a)-(b)). Scale bar represents 200 *μ*m.

Light microscopy images were grabbed utilizing a Nikon Eclipse 55i microscope equipped with a 5-megapixel Nikon DS-Fi1 CCD cooled color camera, together with the Nikon NIS-Elements Basic Research image analysis software (Nikon, Apidrag, Bucharest, Romania). Three investigators (AS, CM, and DP) followed the sections individually and, based on the nuclear glandular-like histological staining and the immunohistochemistry for basal cells, respectively, collected suggestive images for Gleason gradings 2, 3, 4, and 5 as well as AAH and benign nodular hyperplasia (BNH), according to the latest WHO grading system. Images have been collected with a 40x objective, either as single frames (57.6583,69 *μ*m^2^) or as composites of 24 400x objective areas automatically merged as unique captures in the Nikon NIS-Elements software (Figures 1 and 2). More than 500 images have been captured, saved, and archived as uncompressed tiff files. After confirming once again the grading of the captured images and removing all images on which the investigators did not agree (AS, DP, and CM), AgNOR dots were first manually counted and averaged for epithelial nuclei, using the manual tag option in our image analysis software.

Next, nucleolar organizers have been selected as regions of interests (ROIs) and subtracted as binary images from the original RGB images, based on their color profile, intensity, texture, background, and morphological filters found under the “smart segmentation” command in the Image-Pro-Premier image analysis software package (trial version, Media Cybernetics, Bethesda, MD, USA) (Figures 1 and 2).

**Figure 3:**
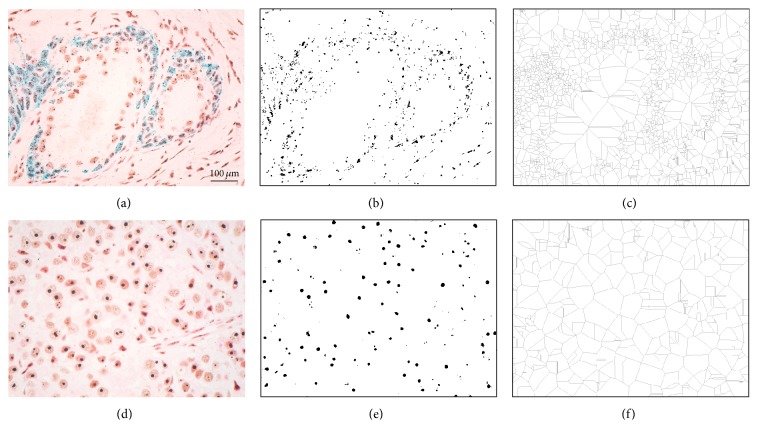
Example of binarisation and pruning filtering. After segmentation of original images ((a), (d)), argyrophilic nucleolar organizer regions (AgNORs) are binarised ((b), (e)) and then skeletons of the AgNOR dots are extracted ((c), (f)) in order to offer a quantifiable view of the glandular distribution of the dots. Scale bar represents 100 *μ*m.

Moreover, in order to better address also the morphology and the relationships between nucleolar organizers rather than the individual ROIs, the binary images have also been processed through a pruning morphological filter reducing the images to their skeletons (Figure 3). All final images were analyzed regarding their fractal dimension, utilizing the same approach in Image-Pro-Premier.

### 2.4. Statistical Analysis

All the data were represented graphically and further analyzed utilizing Microsoft Excel 2010 and SPSS 10.0 (SPSS Inc., Chicago, IL, USA). All areas and roundness values equal to 0 and FDs equal to 1 have not been considered in this analysis in order to filter out smaller, unequivocally stained particles. All measured values were averaged for each image, patient, and gradings and have been compared utilizing a one-way ANOVA with the Tukey correction as post hoc analysis. Pearson testing was utilized to explore correlations, and, in all cases, *P* < 0.05 was used to indicate statistical significance.

## 3. Results

After segmenting the nucleolar organizer regions, we have looked at multiple individual and integrative morphological parameters in order to evaluate the possibilities of stratification of the different pathological entities.

**Figure 4:**
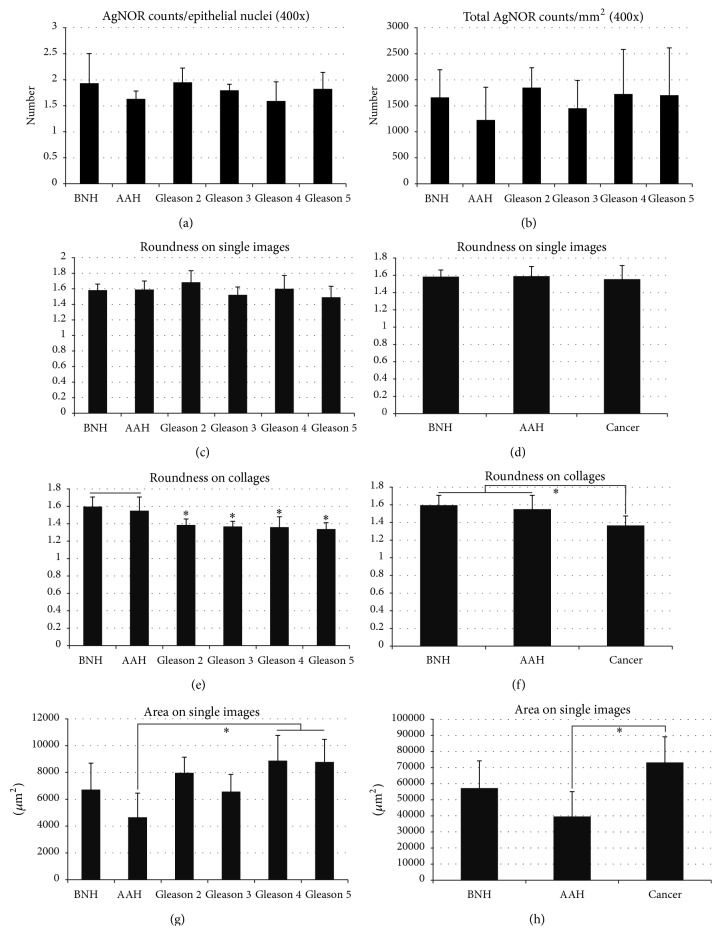
Morphometric assessment of argyrophilic nucleolar organizer regions (AgNORs). AgNORs, counted as average per epithelial cells' nuclei (a) or without discrimination for all the cells in the 40x area (b), show no difference between different pathologies. Roundness factor of the AgNOR dots on individual 40x images cannot account also for difference between pathologies ((c), (d)). Roundness factor of the AgNOR dots on collages, however, could differentiate each Gleason pattern from benign nodular hyperplasia (BNH) and atypical adenomatous hyperplasia (AAH) ((e), (f)). Averaged areas of AgNORs have a very limited value, being able to differentiate only AAH from Gleason patterns 4 and 5 ((g), (h)). Bars represent standard deviation. *∗* represents significance on corrected ANOVA testing.

First of all, we have counted the individual nucleolar organizer regions as stained and segmented by our image analysis algorithm. The number of silver stained dots was first evaluated as an average per glandular epithelial cell nucleus, and although these averages tended to show higher values for Gleason patterns 4 and 5, they did not exhibit any statistical separation power (*P* > 0.05) (Figure 4(a)). Next, we looked at the total densities of AgNOR entities on both 40x individual images (Figure 4(b)) and composed collages, thus without separating epithelial cells from the stromal component (data not shown). AAH tended to show a somehow lower density of stained ROIs, but this difference was again not significant for both individual Gleason scores overall cancers pooled together versus benign conditions (*P* > 0.05) (Figure 3(b)).

Roundness analysis on high resolution images seemed to offer lower global values (1.554 ± 0.158) for carcinoma compared to AAH (1.589 ± 0.110) and nodular benign hyperplasia (1.583 ± 0.077), although the high intracase variability leads to no statistical differences (Figures 4(c) and 4(d)). On composed images, however, acinar adenocarcinoma showed clearly lower values compared to AAH and BNH (*F*(5,54) = 5.887, *P* < 0.001; with post hoc comparisons using the Tukey HSD test (*P* < 0.01)) (Figure 4(e)). Also, carcinoma cases pooled together (1.365 ± 0.107) showed clear-cut lower roundness values compared to atypical adenomatous hyperplasia (1.549 ± 0.248) and nodular benign hyperplasia (1.596 ± 0.109) (*F*(2,54) = 13.173, *P* < 0.001; with post hoc comparisons using the Tukey HSD test (*P* < 0.01)) (Figure 4(f)). No significant differences could be found between any individual Gleason grading, AAH, and BNH on either single 40x images or composites.

A one-way ANOVA found a global difference between the total normalized areas of AgNOR positive pixels for different gradings (*F*(5,40) = 5.396, *P* < 0.001), and post hoc comparisons indicated a difference only between AAH (545.91 ± 210.94) compared to Gleason grading 4 (1041.43 ± 221.94, *P* < 0.01) and, respectively, AAH compared to Gleason grading 5 (1030.89 ± 196.93, *P* < 0.01) (Figure 4(g)). Altogether, carcinoma cases (8592.98 ± 1723.62) showed higher total pixel areas compared to atypical adenomatous hyperplasia (4653.73 ± 1798.22) and nodular benign hyperplasia (6715.97 ± 1983.22) (*F*(2,40) = 11.820, *P* < 0.001; with post hoc comparisons (*P* < 0.05)) (Figure 4(h)). The same trend was also identified for collage images (data not shown).

**Figure 5:**
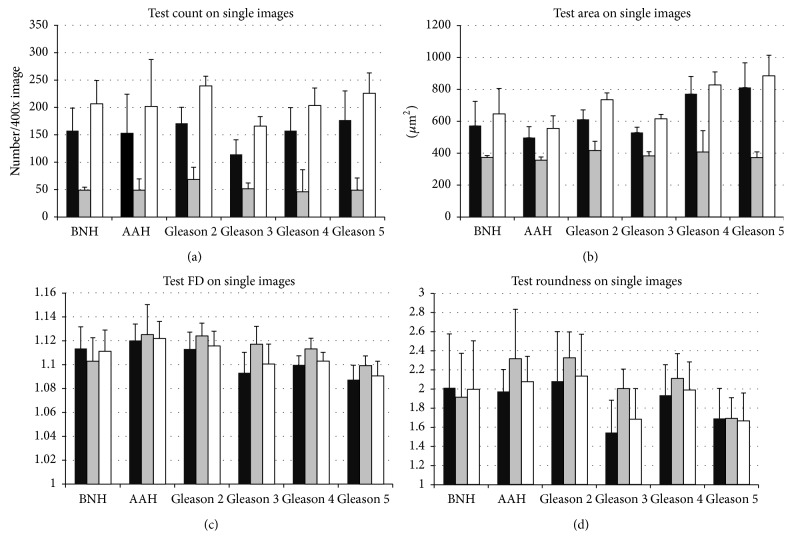
Testing different measurements for argyrophilic nucleolar organizer regions (AgNORs) for select epithelial, stromal, and complete 400x areas showed strong direct correlations between data coming from epithelia and complete tissue areas (*r* > 0.89 on Pearson testing).

Besides the fact that analysis of complete images will add more objectivity rather than separating the glands only, we also assessed whether there was a correlation between the values for direct AgNOR counting, areas, roundness, and FD on a select set of images analyzed for epithelia, stroma, and complete areas (Figure 5). The result was that, for all approaches, epithelia variations could predict very closely overall tissue variations (*r* > 0.89) compared to stroma-complete tissue variations (*r* < 0.64).

**Figure 6:**
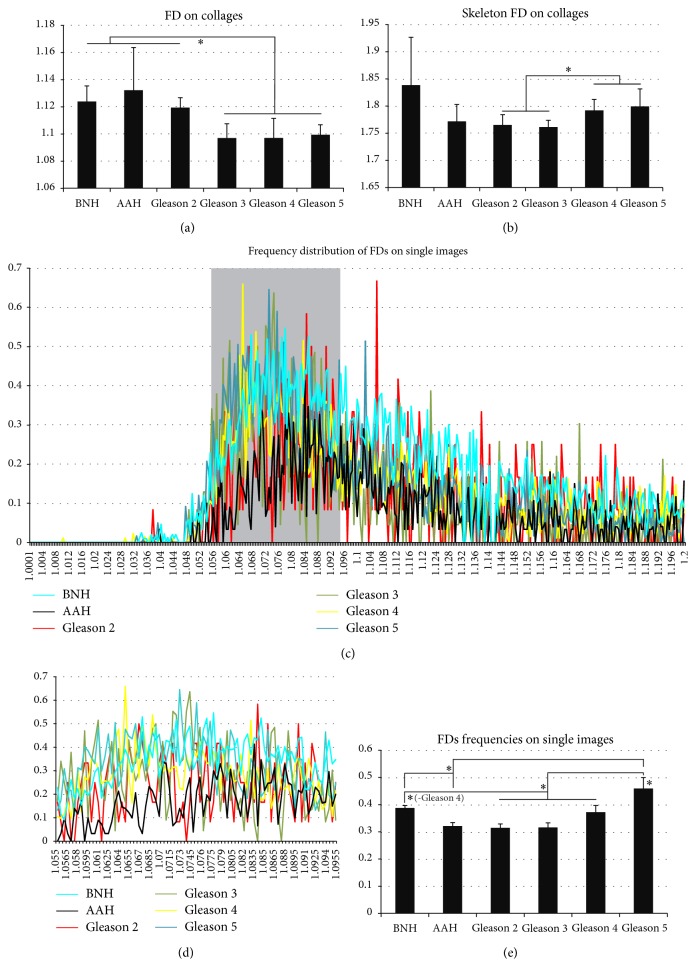
Fractal analysis assessment of argyrophilic nucleolar organizer regions (AgNORs). Benign nodular hyperplasia (BNH), atypical adenomatous hyperplasia (AAH), and Gleason pattern 2 have significantly higher fractal dimensions (FDs) compared to Gleason patterns 3–5 (a). Skeleton reductions of the AgNORs could show significant differences only between Gleason patterns 2-3 group and Gleason patterns 4-5 group (b). Frequency distribution of FDs reveals global maxim values for the interval of 1.05–1.09 (c), and after plotting the average values for this narrowed interval, the filtered data revealed significant differences between BNH, AAH, Gleason patterns 2–4 group, and Gleason pattern 5 group ((d)-(e)). Bars represent standard deviation ((a), (b)) or standard errors of the means (e). *∗* represents significance on corrected ANOVA testing.

We next looked at the averages of fractal dimensions of the silhouettes of nucleolar organizer regions (Figure 6(a)). On individual Gleason scoring, this approach revealed clear-cut differences between Gleason 2 (1.119 ± 0.0007) and Gleason 3–5 group (1.096 ± 0.010; 1.097 ± 0.014; 1.099 ± 0.007), as well as between Gleason 3–5 group and AAH (1.132 ± 0.031) and, respectively, BNH (1.123 ± 0.011) (*F*(5,54) = 8.492, *P* < 0.001; with post hoc comparisons (*P* < 0.05)). Upon grouping together the cases of acinar adenocarcinoma (1.101 ± 0.013), these were deemed lower than both AAH and BNH (*F*(2,54) = 14.999, *P* < 0.001; with post hoc comparisons (*P* < 0.01)) (data not shown).

In order to evaluate as much as possible the morphological architecture of the glandular distribution of the silver dots, we have next performed a fractal analysis of the skeleton-filter images extracted from the repartition of the nucleolar organizers. Gleason 2 and 3 patterns (1.765 ± 0.018; 1.1761 ± 0.012) were lower than Gleason 4-5 patterns (1.792 ± 0.020; 1.1799 ± 0.032) (*F*(5,54) = 4.552, *P* < 0.01; with post hoc comparisons (*P* < 0.05)) (Figure 6(b)). Overall, carcinoma cases could not be differentiated from AAH and BNH utilizing this classifier only.

In a last attempt to evaluate the ruggedness of the silhouettes of nucleolar organizers, we have plotted the frequency distribution of the averaged fractal dimensions of the AgNORs from the individual high magnification images and collages (Figure 6(c)). The total data revealed no global significant differences between the seven pathological entities considered on ANOVA testing for individual images (*F*(5,1456) = 2.214, *P* = 0.051) and, respectively, for composites (*F*(5,1435) = 1.388, *P* = 0.227). Observing that the relative peaks of the distributions were concentrated in the 1.05–1.09 FD interval values, we have next analyzed only the data coming from this narrowed interval (Figures 6(d) and 6(e)). Narrowed data from composed images did not exhibit a great deal of differences (*F*(5,3395) = 4.443, *P* = 0.001), with post hoc comparisons revealing a significant difference only between Gleason 4 and AAH (*P* < 0.001) (data not shown). Narrowed data from individual images, however, revealed not only a global difference between the trends (*F*(5,3184) = 19.832, *P* < 0.001), but also significant differences between all Gleason stages and BNH, although AAH could be clearly differentiated only from Gleason 5 cases (post hoc comparisons, *P* ≤ 0.05) (Figures 6(d) and 6(e)).

## 4. Discussion

Morphological features of AgNOR, as depicted by silver impregnation techniques, have been utilized to compare cancers with normal structures and nonmalignant neoplasia [29, 30]. For prostate cancer, besides algorithms that attempted to recognize glandular morphology in order to identify malignant prostate tissue areas, AgNOR silver impregnation has been utilized in various studies based on manual, semiautomatic, or fully automatic scoring methods, taking into account the number, aggregation, and size of argyrophilic dots in relation to clinicopathological prognostic parameters for prostatic carcinoma [13–16].

Our study was performed on a group of 39 cases that included BNH, AAH, and adenocarcinomas of the prostate, for which we have analyzed the number, roundness factor, area, and fractal dimension of individual AgNOR signals or of their skeleton reductions. In different other studies, the number of AgNOR signals provided significant [14, 15, 21, 29] or insignificant [30, 31] differences for malignant lesions of the prostate, with significantly higher values for high Gleason scores [14, 15, 21]. Compared to the direct counting of AgNOR signals, morphometric analysis allows a more objective and reproducible quantification on histological sections [17, 32].

In our study, AgNOR direct counting and averaging per nucleus of epithelial cells revealed no significant difference between the pathologies. This difference from studies finding to some extent differences between pathologies could be due to different issues, namely, the different histological techniques and assessment protocols. First, we have implemented here a standardized AgNOR staining, as recommended by the “International Committee on AgNOR Quantitation” [12]. On the other hand, in the present study, we have also performed a selective count only in the nuclei of luminal glandular cells, based on combined silver staining and basal cells immunohistochemistry. Moreover, morphometric analysis is recommended and is based on a common fixed threshold applied to all images to be segmented, resulting in more objective and constant results [33, 34].

Further Euclidian classifiers have been utilized for the differentiation and characterization of malignant lesions of the prostate based on AgNORs analyses, such as their average diameter, total area, or percentage area from the total area of the nucleus [17, 29]. In our study, the analysis of roundness (diameters ratio) and the areas of the AgNORs averaged for large composite images revealed significantly lower values in carcinomas compared to benign and atypical lesions. Both roundness and areas showed no differences for different Gleason scores, issues that are also supported by other published data [29]. In other studies, the analysis of the AgNOR diameters and areas, respectively, indicated significant differences both between benign, atypical, and malignant prostatic lesions and for different Gleason scores, clinical stage, and ploidy of the tumors [17]. All the morphological analysis was done here on all the cells present in the captured images, thus without the need of discriminating glands from parenchyma, greatly increasing the simplicity of the approach and eliminating any user interference that might be necessary to reliably select epithelial tissue from stroma. Also, by utilizing high resolution large composite images, this gave a much more homogenous and reliable approximation of the pathology compared to single high resolution images that might focus on areas with a lower morphological variation.

A relative recent published paper based on utilizing complex in-house software for automatic grading of prostate carcinomas reported correct classification rates of over 90% after employing fractal analysis of the intensity variations and texture complexity of the ROIs [35]. In our study we have both characterized the averaged individual FDs of AgNOR silhouettes and extracted the FDs of skeletonized AgNOR regions which reflected the general glandular disposition of the dots. To our knowledge, this is the first study to perform fractal analysis of silver impregnated nucleoli in prostate pathology [28, 36–38]. Fractal analysis of AgNOR silhouettes regularity not only revealed significant lower values for global cancer images compared to AAH and BNH images, but was also able to differentiate between Gleason 2 group and Gleason 3–5 group of carcinomas. Coupled with the fact that roundness alone could separate cancer samples from AAH and BNH, this leads to an increased selectivity for the combined use of the two denominators. Although this precise methodology has never been employed in the study of nucleolar organizers for prostate cancer, the increasing size and regularity of the nucleoli are a long-standing subjective observation in these tumors [4, 5, 39]. Moreover, a great variability exists regarding the association of large, multiple, and relatively round nucleoli in advanced Gleason stages of prostatic acinar adenocarcinoma [5, 36]. Our data sheds more light on the global morphological changes of nucleoli during prostate cancer progression, revealing that in fact the global tendency of nucleoli is to increase in size and to become less round and with a decreasing complexity of their boundaries, thus coining that the better description of their appearance would be of smoother ellipsoids or ovals rather than circles. Regarding the glandular repartition of the AgNORs, FD of skeleton images revealed significant lower values for carcinomas with Gleason score of 2-3 compared with those with a score of 4-5, but without being able to differentiate global malignant and hyperplastic lesions. However, if we looked again at averaged ROIs areas, FDs and skeleton FDs taken together, the added value is that we can separate almost all pathological states except BNH from AAH and Gleason 4 from 5.

Lastly, after plotting the frequency distribution of the FDs for different pathologies and observing that most of the values are in fact gathered in the interval of 1.05–1.09, we have compared the frequency distribution of FDs only for this narrow interval for all pathologies. Surprisingly, this could differentiate between Gleason 2, 3, and 4 group and Gleason 5, AAH, and BNH group. Most probably this limitation of separating Gleason 5 from AAH and BNH lies somehow in the influence of stromal cells which we have not separated in our algorithm; altogether averaged ROIs areas, FDs, and frequencies of FDs should allow an almost complete separation of each Gleason score from AAH and BNH.

There have been attempts to develop both texture-based [40–42] and fractal-based [35, 43] image analysis algorithms to automate the diagnosis of prostatic carcinoma. Most of these studies were based on color and texture morphological features of glandular and nuclear structures to characterize tissues. The introduction of neural networks and digital mapping led to increased rates of consensus for grading of prostatic carcinoma and achieving good quantification results [44–47]. In the direction of automated image analysis, AgNOR staining may be of interest and may contribute as an implementable parameter among other classifiers in existing image diagnostic software packages.

## 5. Conclusions

Based on a relatively simple staining technique, the present study presents the combined analysis of AgNORs roundness, averaged FDs, and FDs' frequency analysis as a suite of denominators able to differentiate between malignant and nonmalignant lesions of the prostate, as well as between different Gleason scores. Together with existing classifiers already in use such as nuclear, glandular, stromal, and other architectural features, these present data might contribute to faster and more reliable objective additive diagnosis tools of prostatic carcinoma and decreased reports of uncertain histological lesions, as an essential aid for pathologists.
